# Caucasians with acral lentiginous melanoma have the same outcome as patients with stage- and limb-matched superficial spreading melanoma

**DOI:** 10.1007/s00432-021-03630-6

**Published:** 2021-04-15

**Authors:** Laura Susok, Thilo Gambichler

**Affiliations:** Department of Dermatology, Skin Cancer Center, Ruhr-University Bochum, Gudrunstrasse 56, 44791 Bochum, Germany

**Keywords:** Acral lentiginous melanoma, Superficial spreading melanoma, Progosis, Overall survival

## Abstract

**Purpose:**

Acral lentiginous melanoma (ALM), a relatively rare subtype of cutaneous melanoma, has been reported to have a worse prognosis than other melanomas. We aimed to assess clinical findings in Caucasian ALM patients and compare the data with a matched cohort of superficial spreading melanoma (SSM) patients.

**Methods:**

We studied 63 patients with ALM and 63 randomly stage- and limb-matched patients with SSM (non-ALM). In both cohorts, guideline-adjusted diagnosis, treatment and follow-up were performed.

**Results:**

We did not observe differences in prognostic factors (e.g., tumor thickness, ulceration) between the two cohorts. Both in ALM and non-ALM patients positive sentinel lymph node was a significant independent predictor for disease relapse and melanoma-specific death. However, disease relapse and melanoma-specific death rates did not significantly differ between ALM and non-ALM patients. An overall 5-year melanoma-specific survival of 82.5% and 81% was observed in ALM and non-ALM patients, respectively.

**Conclusions:**

Our data confirm that patients with ALM have no worse outcome than non-ALM patients when correcting for significant prognostic factors. Hence, the reportedly high rates of fatal ALM cases should not be ascribed to pathobiological differences between ALM and non-ALM but are most likely are a consequence of a delay in diagnosis and thus advanced stage of ALM.

## Introduction

Acral lentiginous melanoma (ALM), occurring on the hands and feet (palms, soles, fingers, toes, and nail units) was first defined as a distinct subtype of cutaneous melanoma (CM) by Arrington et al. (1977). It is the least common subtype of CM comprising only about 2.5% of total melanoma diagnoses in Caucasians. Unlike in other CM subtypes, ultraviolet radiation does unlikely play a significant role in the development of ALM, which is rather a low mutational burden CM subtype (Hall and Rapini 2020). Mutations in ALM particularly include the KIT gene in over 30% of ALM cases. Histopathologically, ALM most frequently shows a radial growth phase in which the lesion remains in-situ. The invasive portion of ALM usually resembles superficial spreading melanoma (SSM) and may secondarily develop a nodular growth pattern (Arrington et al. 1977; Basurto-Lozada et al. 2021; Fernandez-Flores and Cassarino 2017; Hall and Rapini 2020).

ALM is most commonly located on the lower limbs and has a much higher proportional prevalence in non-Caucasians, such as Hispanics (over 40%), Asians (over 50%), and Africans (over 60%) (Hall and Rapini 2020; Duarte et al. 2017; Lino-Silva et al. 2019; Lv et al. 2016). ALM is often advanced at the time of presentation resulting in a high level of morbidity and mortality (Hall and Rapini 2020; Lino-Silva et al. 2016). When compared to other CM subtypes ALM appears to have a significantly worse prognosis (Duarte et al. 2017; Lv et al. 2016; Bello et al. 2013). We aimed to assess clinical prognostic features in Caucasian ALM patients and compare them with a random stage- and limb-matched cohort of SSM patients.

## Materials and methods

### Patients

Patients’ data were extracted from a large institutional research database of CM patients (*n* = 1936). All patients had been treated at the Skin Cancer Center of the Department of Dermatology (Ruhr-University Bochum) between January 2001 and May 2011. The study population extracted from the database included 63 patients with invasive ALM (Table 1). Patients with in-situ melanomas were not included in the analysis. Patients’ data, including gender, age, tumor thickness, ulceration, regression etc., were collected from the electronic records. Patients were staged or re-staged according to the final version of the 2009 AJCC melanoma staging and classification system (Balch et al. 2009). Following the data extraction of all ALM cases, we selected invasive SSM (non-ALM) patients as follows: ALM patients were matched to non-ALM patients with SSM on the lower extremities or upper extremities who were listed in the research database. Patients with non-ALM and aforementioned characteristics were randomly selected by means of the web-based random thing picker software by Hedges (2021).

**Table 1:** Comparison of acral lentiginous melanoma (ALM) patients (*n* = 63) with randomly stage—limb-matched patients (*n* = 63) with superficial spreading melanoma of the lower or upper extremities (non-ALM)

Parameters	Non-ALM *n* = 63	ALM *n* = 63	*P*-value (Mann–Whitney and Chi^2^ test)
Age (median (range) years)	67 (19–91)	69 (17–93)	=0.15
Gender (f/m)	46/17 (73%/27%)	36/27 (57.1%/42.9%)	=0.48
Location (upper/lower limbs)	7/56 (11.1%/88.9%)	7/56 (11.1%/88.9%)	n.a
Clark level			=0.78
II	3 (4.8%)	7 (11.1%)	
III	19 (30.2%)	6 (9.5%)	
IV	38 (60.3%)	44 (69.8%)	
V	3 (4.8%)	6 (9.5%)	
Tumor thickness (mm)	1.4 (0.4–7)	1.6 (0.2–15)	=0.47
High-risk melanoma (>2 mm thickness)			
No/yes	39/24 (61.9%/38.1%)	36/27 (57.1%/42.9%)	=0.88
Regression			
No/yes	57/6 (90, 5%/9.5%)	59/4 (93.7%/6.3%)	=0.59
Ulceration			
No/yes	43/20 (68.3%/31.7%)	42/21 (66.7%/33.3%)	=0.13
Nodal nevus			
No/yes	59/4 (93.7%/6.3%)	63/0 (100%/0%)	<0.0001
Adjuvant interferon			
No/yes	38/25 (60.3%/39.7%)	39/24 (61.9%/38.1%)	=0.10
Disease relapse			
No/yes	45/18 (71.4%/28.8%)	43/20 (68.3%/31.7%)	=0.87
5-year melanoma-specific death			
No/yes	51/12 (81%/19%)	52/11 (82.5%/17.5%)	=0.97

*n.a.* Not applicable (predefined)

All primary tumors were examined by at least two senior dermato-histopathologists of the Skin Cancer Center of the Department of Dermatology (Ruhr-University Bochum). Immunohistochemistry was carried out with antibodies against S100B and Melan-A/MART-1 and in ambiguous cases also with HMB45 and Ki-67 (DAKO, Hamburg, Germany). The management of patients was guideline-adjusted according to the tumor stage (Balch et al. 2009; Garbe et al. 2006). All tumors were diagnosed and treated by primary excision including safety margin if indicated. Predominant indication for sentinel lymph node biopsy (SLNB) was a Breslow tumor thickness of 1 mm or more. Upgrading of tumors less than 1 mm was considered in the presence of a Clark level of IV or higher and ulceration. Prior to SLNB, evidence of macro-metastatic disease in regional lymph nodes or distant sites was ruled out by physical examination and imaging with computed tomography. Patients with metastatic regional lymph nodes were subjected to complete lymph node dissection. All patients with a primary melanoma thickness of 1.5 mm or more were considered for adjuvant low-dose interferon alfa-2b (Roferon; Roche Pharma AG, Grenzach-Wyhlen, Germany) therapy, and patients with melanoma-positive lymph nodes were considered for adjuvant high-dose interferon (Intron; MSD, Munich, Germany) therapy. Metastatic disease was usually treated with mono-dacarbacine or -temozolomide or polychemotherapy using gemcitabine and treosulfan or carboplatin and paclitaxel (Garbe et al. 2006). Follow-up data were collected using chart review and contacting patients, relatives, and resident practitioners and dermatologists. The study was approved by the Ethics Committee of the Ruhr-University Bochum (#4749-13) and conducted according to the principles of the Declaration of Helsinki.

### Statistics

Data analysis was performed using the statistical package MedCalc Software version 19.8 (MedCalc, Ostend, Belgium). Distribution of data was assessed by the D’Agostino-Pearson test. For non-normally distributed data, the median and range were calculated. Data were analyzed using the Mann–Whitney test, Chi-square test, Kaplan–Meier curves including the log-rank test, and Cox proportional-hazards regression analysis. *P*-values smaller than 0.05 were considered significant.

## Results

At the time of primary diagnosis, ALM patients and stage- limb-matched non-AML patients were in the stage as follows: IA = 15 (23.8%), IB = 12 (19%), IIA = 4 (6.3%), IIB = 13 (20.6%), IIC = 1 (1.6%), IIIA = 8 (12.7%), IIIB = 8 (12.7%), IIIC = 2 (3.2%). As demonstrated in Table 1, age (*P* = 0.15), gender (*P* = 0.87), Clark level (*P* = 0.78) tumor thickness (*P* = 0.47), Breslow thickness > 2 mm (*P* = 0.88), regression (*P* = 0.59), ulceration (*P* = 0.13), and adjuvant treatment with interferon (*P* = 0.10) did not significantly differ between the ALM and non-AML cohort. However, the presence of nodal nevi on SLNB was significantly (*P* < 0.0001) associated with non-ALM. SLNB status and subsequent complete lymph node dissection procedures did not significantly (*P* = 0.93 and *P* = 0.41, respectively) differ between AML (17 positive cases/15 procedures) and non-AML (18 cases/17 procedures).

**Fig. 1:**
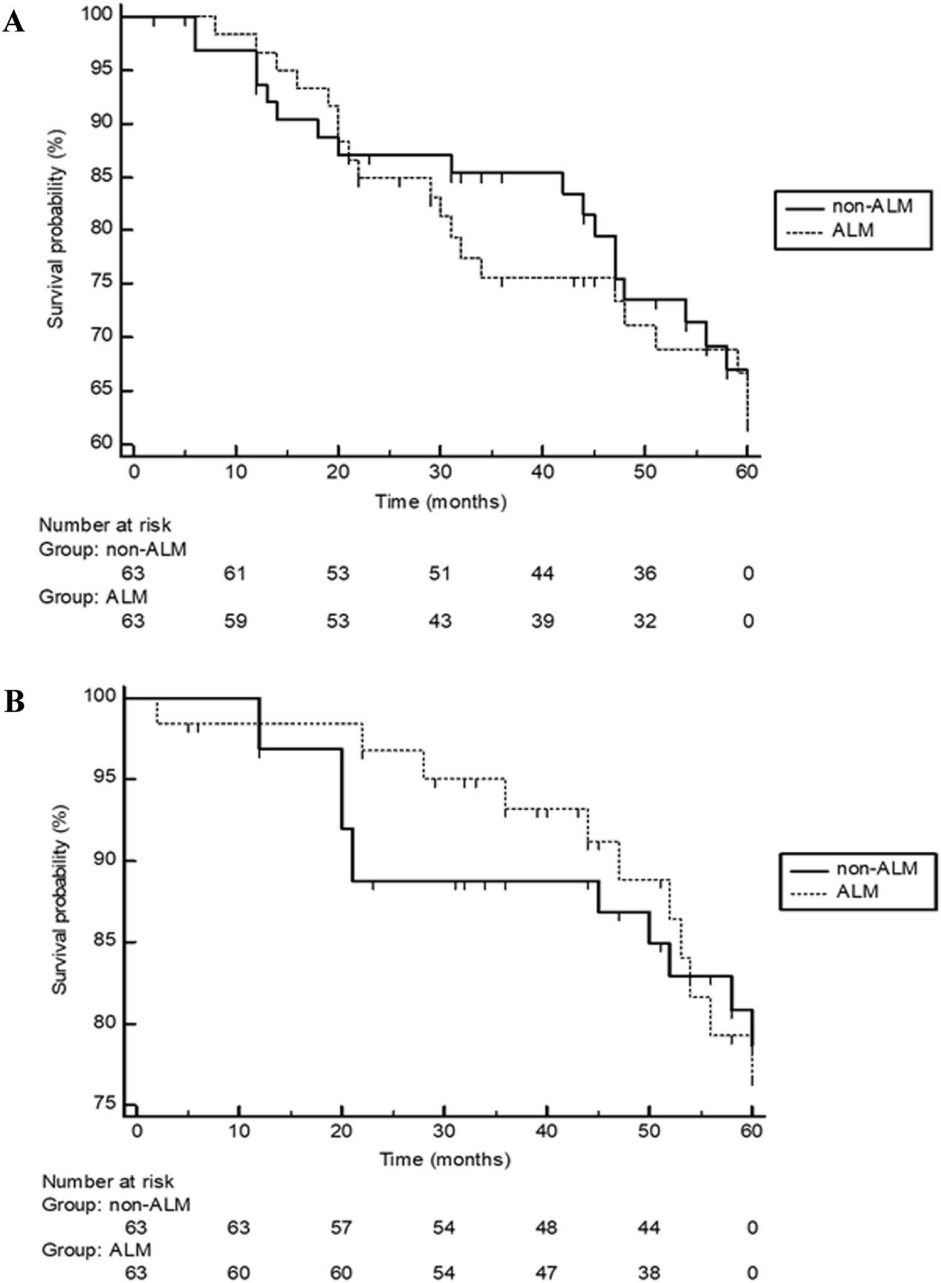
Showing the 5-year disease relapse Kaplan–Meier curves of Caucasian patients with acral lentiginous melanoma (ALM; *n* = 63) and randomly stage—limb-matched patients (*n* = 63) with superficial spreading melanoma of the lower and upper extremities (non-ALM). Disease relapses did not significantly (log-rank test: *P* = 0.62) differ between both groups (**a**). Moreover, melanoma-specific deaths did not significantly (log-rank test: *P* = 0.97) differ between both groups (**b**)

The median time to disease relapse was 51 months (2–60) in ALM and 56 months (6–60) in non-ALM; median melanoma-specific survival was 60 months (2–60) for ALM patients and 60 months (12–60) for non-ALM patients. Disease relapse rates and melanoma-specific death rates did not significantly differ between ALM patients and non-ALM patients (*P* = 0.87 and *P* = 0.93, respectively; Fig. 1a,b). Disease relapse was more likely observed in ALM (*P* = 0.016) as well as non-ALM (*P* = 0.018) patients who had a primary melanoma thicker than 2 mm. Unlike non-ALM patients, ALM patients with a primary thicker than 2 mm more likely died from melanoma (*P* = 0.029). On Cox proportional-hazards regression, however, positive SLNB status was the only significant prognostic factor for melanoma-specific death in ALM (*P* = 0.049) as well as non-ALM (*P* < 0.0001).

## Discussion

Several previous investigations revealed that ALM represents a very aggressive CM subtype (Bello et al. 2013; Csányi et al. 2020; Duarte et al. 2017). The question is, however, whether ALM really represents a biologically more aggressive CM subtype per se or whether the worse outcome observed in this melanoma subset is just due to delayed diagnosis and advanced stage at first presentation? The long delay with a duration sometimes over years in the diagnosis of ALM was described in most previous papers (Lv et al. 2016). A lot of factors apparently contribute to the delay in diagnosis, including indolent elderly, hidden site, lack of pigmentation, lack of recognition and misdiagnosis by physicians (Lv et al. 2016). ALM is frequently misinterpreted and thus mismanaged as callus or warts by practitioners, resulting in a considerable delay of the correct diagnosis and appropriate treatment. In contrast to Caucasians (about 2 mm), Asians, Hispanics, and Blacks initially present with Breslow tumor thickness often higher than 4 mm. The difference between tumor thickness at diagnosis in different ethnic groups may also explain the heterogeneity in disease outcome. Similar as observed in our population, Caucasians with ALM usually have a 5-year overall survival rate of about 80%, whereas the rates in Chinese (about 50%) and Hispanics (about 50%) are much worse which can be explained by idiosyncratic, socioeconomic, infrastructural, and geographic issues in these countries (Bradford et al. 2009; Behbahani et al. 2021; Lino-Silva et al. 2019; Lv et al. 2016).

Indeed, many research groups have observed that overall survival rates of ALM patients are lower when compared to patients with SSM alone or all melanoma subtypes, except for ALM (Bello et al. 2013; Csányi et al. 2020; Duarte et al. 2017; Huang et al. 2020; Kuchelmeister et al. 2000; Lv et al. 2016; Phan et al. 2006; Slingluff et al. 1990). Using SSM as the reference category, El Sharouni et al. (2020) observed adjusted hazard ratios of 1.26 (95% CI 1.06–1.50) for ALM. Mejbel et al. (2021) retrospectively analyzed the clinicopathologic factors of thin ALM (≤1 mm; *n* = 129) and thin non-ALM (*n* = 699). Their data suggest that ALM is inherently more aggressive than other types of melanoma. In multivariable analysis, Mejbel et al. (2021) found that ALM histologic type, positive SLN status, age, and the use of systemic therapy were detected as independent poor prognostic factors associated with significantly lower survival rates. Recently, Howard et al. (2020) studied 101 ALM patients demonstrated poorer melanoma-specific rates of patients with ALM when compared to SSM. Since SSM accounts for the most melanoma cases in Caucasians and shares histopathological features of ALM, we aimed to compare these two CM subtypes with respect to clinical outcome using a stage–limb-matched approach. Regarding important prognostic factors such as tumor thickness, ulceration and SLNB status, both groups were homogenously distributed and thus comparable with each other. We did not observe differences in outcome measures, including disease relapse and melanoma-specific death, between ALM and non-ALM. Breuninger et al. (1994) previously compared ALM with matched SSM of high-risk locations such as head/neck and genital region. Similar to our data the 5-year Kaplan–Meier curves were almost identical in both groups. Hence, Breuninger et al. (1994) concluded that the poor prognosis frequently ascribed to ALM results from the prognostic factor location. In a Turkish population, Tas and Erturk (2018) compared ALM (*n* = 102) with other melanoma subtypes and did not find correlations between ALM in either nodal involvement or distant metastasis. The disease-free survival and overall survival rates for ALM patients were similar to those of patients with other histological subtypes (Tas and Erturk 2018). Similar to our study, Lino-Silva et al. (2019) performed a stage-matched analysis of ALM (*n* = 715) and non-ALM (*n* = 429) patients in Mexico. However, they did not adjust for tumor location as we did. They observed a disease-specific survival of only 46% in ALM patients and 55.7% in non-ALM patients, which was not statistically significant (*P* = 0.16; Lino-Silva et al. 2019). Hence, our outcome data ALM patients obtained from a Caucasian cohort is in line with this large comparative study on Hispanics (Lino-Silva et al. 2019). Like other CM subtypes, melanoma-specific survival in ALM is affected by gender, race, age, Breslow thickness, ulceration, pathologic stage, and sentinel lymph node positivity (Hall and Rapini 2020). The discrepancies reported in the literature regarding survival data of ALM patients may also be due to differentially reported outcome measures, for example, overall survival vs. melanoma-specific survival. Importantly, this is of interest as ALM is associated with high age. Accordingly, Eriksson et al. (2015) prospectively studied 4237 melanoma patients of all tumor subtypes and did not observe that melanoma-specific death rates differ between ALM and SSM patients. As also observed in the present study, positive SLNB is the strongest predictor of disease relapse and melanoma-specific death (Wei et al. 2020). Similar to our approach, Huayllani et al. (2020) compared a large sample of AML (*n* = 5203) with patients with melanomas located on the limbs, exclusively ALM (Huayllani et al. 2020). When compared with patients with melanomas located on the limbs, those with ALM were more likely to be older than 80 years at diagnosis, regional lymph node metastases, and have ulceration. Unfortunately, they did not provide survival data (Huayllani et al. 2020).

Another interesting finding of the present study is that nodal nevi (6.3%) were only observed in non-ALM patients. Indeed, nodal nevi are found in 0.12% to 0.54% of nodes from complete lymph node dissections, 1.2% of nodes from selective lymph node dissections, and about 4% to 13% of sentinel lymph nodes (Gambichler et al. 2013). We have previously demonstrated in 50 melanoma patients with nodal nevi that a primary on the lower limbs is a strong independent negative predictor of nodal nevi (Gambichler et al. 2013). Based on our present findings the aforementioned observation may be explained by the presence of ALM. By contrast, Kim et al. (2018) showed in an Asian population that the prevalence of nodal nevi in ALM was similar to that reported in Caucasians. Hence, ethnic differences may play a role as well.

In conclusion, ALM patients often present at a more advanced stage, which is thought to be multifactorial in cause, including socioeconomic and cultural factors (Behbahani et al. 2021; Hall and Rapini 2020). However, our data demonstrate that Caucasian patients with ALM have no worse outcome than non-ALM Caucasians when adjusting for significant prognostic factors such as disease stage and primary location. Hence, the frequently reported high rates of fatal ALM cases should not mainly be ascribed to pathobiological differences between ALM and non-ALM but are most likely a consequence of delay in diagnosis and thus advanced stage of ALM.
